# The BeUpstanding Program™: Scaling up the *Stand Up Australia* Workplace Intervention for Translation into Practice

**DOI:** 10.3934/publichealth.2016.2.341

**Published:** 2016-05-30

**Authors:** Genevieve N Healy, Ana Goode, Diane Schultz, Donna Lee, Bell Leahy, David W Dunstan, Nicholas D Gilson, Elizabeth G Eakin

**Affiliations:** 1 The University of Queensland, School of Public Health, Brisbane, Queensland, Australia; 2. Baker IDI Heart & Diabetes Institute, Melbourne, Victoria, Australia; 3. School of Physiotherapy and Exercise Science, Curtin University, Perth, Australia; 4. Workplace Health and Safety Queensland, Office of Industrial Relations, Queensland Treasury, Brisbane, Queensland, Australia; 5. School of Public Health and Preventive Medicine, Monash University, Melbourne, VIC, Australia; 6. Department of Medicine, Monash University, Melbourne, Victoria, Australia; 7. School of Exercise and Nutrition Sciences, Deakin University, Burwood, Victoria, Australia; 8. School of Sport Science, Exercise and Health, The University of Western Australia, Perth, Western Australia, Australia; 9. Mary MacKillop Institute for Health Research, Australian Catholic University, Melbourne, Victoria, Australia; 10. The University of Queensland, School of Human Movement Studies and Nutrition Sciences, Brisbane, Queensland, Australia

**Keywords:** workplace, intervention, dissemination, sedentary, activity, translation, sitting

## Abstract

**Context and purpose:**

Too much sitting is now recognised as a common risk factor for several health outcomes, with the workplace identified as a key setting in which to address prolonged sitting time. The *Stand Up Australia* intervention was designed to reduce prolonged sitting in the workplace by addressing influences at multiple-levels, including the organisation, the environment, and the individual. Intervention success has been achieved within the context of randomised controlled trials, where research staff deliver several of the key intervention components. This study describes the initial step in the multi-phase process of scaling up the *Stand Up Australia* intervention for workplace translation.

**Methods:**

A research-government partnership was critical in funding and informing the prototype for the scaled up BeUpstanding program™. Evidence, protocols and materials from *Stand Up Australia* were adapted in collaboration with funding partner Workplace Health and Safety Queensland to ensure consistency and compatibility with existing government frameworks and resources. In recognition of the key role of workplace champions in facilitating workplace health promotion programs, the BeUpstanding program™ is designed to be delivered through a stand-alone, free, website-based toolkit using a ‘train the champion’ approach.

**Key findings and significance:**

The BeUpstanding program™ was influenced by the increasing recognition of prolonged sitting as an emerging health issue as well as industry demand. The research-government partnership was critical in informing and resourcing the development of the scaled-up program.

## 1. Introduction

Changing job roles and job tasks have led to increased opportunities for the office worker to spend much of the workday sitting. Recent estimates suggest that, on average, 75% of the workday is spent in this posture, with much of this time accrued in prolonged unbroken bouts of 30 minutes or more [1], [2]. Moreover, workplace sitting is the largest contributor to daily sedentary time in workers [3]. These high levels of sitting, coupled with the rapidly accruing evidence on the detrimental health impacts of too much sitting [4], [5], has led peak policy bodies to identify too much sitting as an emerging occupational health and safety issue [6], [7]. Associated with this is increased demand from industry for information and programs to address too much sitting in the workplace, evidenced by the commissioning of expert statements [6], [8], and the emergence of active working summits [9], [10]. In the research environment, reductions in workplace sitting have now been successfully achieved within the context of pilot studies and randomised controlled trials [1], [11], [12]. Although a number of advocacy / awareness raising campaigns have emerged (e.g. *Get Britain Standing; On Your Feet Australia*), evidence based programs have not yet been systematically translated into practice.

**Figure 1.**
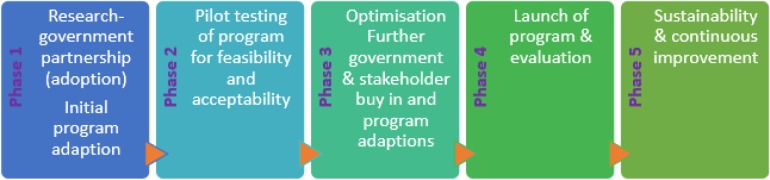
Phases of translation from research into practice for the BeUpstanding program™

One of the interventions that has effectively reduced workplace sitting is the Stand Up Australia intervention [1], [11], [13]. Stand Up Australia is an evidence-guided, workplace-delivered intervention that aims to reduce prolonged sitting [14]. As described below, it is part of the broader Stand Up Australia program of research. This paper describes the initial phase of the multi-phase process of translating the Stand Up Australia intervention for widescale uptake into practice. As outlined in Figure 1, the phases are: Phase 1- Research-government partnership leading to initial adaptations to the Stand Up Australia intervention and the development of a free online toolkit; Phase 2- pilot testing the toolkit in a sample of workplaces for feasibility and acceptability; Phase 3- optimisation and further adaptions of the program and associated toolkit for launch in Phase 4- launch and evaluation; Phase 5- sustainability and continual improvement. The intent of detailing the processes involved with translating the Stand Up Australia intervention (including the initial phases that are often overlooked in reporting) is to build a translational evidence base to inform other research-to-practice efforts in the field.

## 2. Stand Up Australia

The Stand Up Australia program of research aims to understand the benefits of reducing prolonged sitting in the workplace. This program, initiated in 2009, spans across epidemiological [15]–[18], experimental [19]–[21], intervention [1], [11], [22], [23], and translational work. The Stand Up Australia intervention is the flagship intervention of the program. It is a multi-component intervention with strategies intended to address the multiple levels of influence on prolonged sitting, including organisational factors, the physical work environment, and characteristics of individual employees. As described previously in detail [14], this theory-based intervention was systematically developed through iterative pilot testing [1]. Notably, the intervention was based on workplace health promotion best practice [24], [25] and designed with consideration for scale-up and dissemination. The key message of the intervention is to stand up, sit less, move more. These targets aim to reduce sitting time — particularly sitting time accrued in prolonged, unbroken bouts of at least 30 minutes — replacing it with either standing or stepping, and to do this across the whole day (both in and out of the workplace). A participative approach is used, where workplace teams choose, as a group, which strategies they will implement. As part of the intervention, individuals also receive feedback on their current levels of sitting, standing and activity, which is used by health coaches to help set individual goals for the three stand up, sit less, move more targets. Workplace champions (typically the team manager) are also identified. Their task is to reinforce the intervention messages and strategies and engage staff with the program, both through role modelling, and also through delivery of emails.

In 2011, funding was awarded from the Australian National Health and Medical Research Council (APP#1002706) and VicHealth to evaluate the Stand Up Australia intervention. Following a successful pilot [1], the intervention was further evaluated from 2012 to 2014 in a cluster-randomised controlled trial, *Stand Up Victoria* [26], with research staff delivering several of the intervention components (health coaching; facilitating the staff information and brain storming session; sending intermittent emails to team champions for distribution; analysing and providing the activity feedback). The intervention was effective at reducing sitting time both at the workplace and across the day, with an intervention effect of −99.1 (95% CI −116.3 to −81.8) mins/8-h workday and −77.7 (95% CI −100.3 to −55.2) mins/16-h day respectively at the 3-month follow-up [27]. Contact from the research team ceased after this 3 months' follow-up, however, significant intervention effects were still observed one-year post baseline (−45.4 [−64.6 to −26.2] min/8-h workday; −36.3 [−62.6 to −10.0] mins/16-h day) [27]. In addition, the intervention was well received by both managers and staff, and did not impact negatively on productivity [13], as measured by the Health and Work Questionnaire [28].

A key theme arising from the multi-method assessment of the various Stand Up Australia evaluations [1], [11], [13] was the importance of the workplace team champion in promoting the intervention messages and strategies, and creating a supportive organisational culture for change. This finding, consistent with workplace health promotion frameworks [29], strongly informed the ‘train the champion’ approach used to adapt the Stand Up Australia intervention for widescale delivery in collaboration with government partners, as described below.

## 3. Developing a government partnership

In December 2014, the findings from the Stand Up Australia body of work were presented at the annual Australian Workplace Health Promotion conference (Sydney, Australia). Following this presentation, the research team were approached by a representative from Workplace Health and Safety Queensland (WHSQ) Healthy Workers Initiative (HWI). Through the auspices of the Healthier. Happier. Workplaces Initiative, WHSQ were interested in sourcing an evidence based workplace program to address excessive occupational sitting that could be made widely available to workplaces to improve occupational health. The objective of WHSQ is to build the capacity of workplaces to plan, implement and evaluate workplace health and wellbeing programs, manage chronic disease risk factors, as well as address Hazardous Manual Tasks risk factors. As such, the proposed adoption of a program based on the Stand Up Australia intervention directly aligned with WHSQ's objective.

Funding from the Healthier, Happier Workplace Grant Program was successfully obtained through WHSQ in February 2015. Such rapid funding availability, outside the normal grant cycle (where outcomes are typically not known until 8+ months after submission), was a critical element in rapidly progressing the translation process. In addition to the dedicated funding, additional in-kind support (including review of the resources; provision of contacts into industry networks) was provided by WHSQ staff, through the Healthy Worker Inititiave Unit and Ergonomics Unit. Both of these areas had identified excessive occupational sitting as a priority area.

## 4. Adapting the Stand Up Australia intervention for widescale delivery

As part of the process for widescale uptake by workplaces, and to differentiate the program from the research intervention, the Stand Up Australia intervention was rebadged and redeveloped as the BeUpstanding program™. While the core elements of the original intervention remained, several adaptions (described below) were necessary to ensure feasibility and consistency with the best practice Work Health and Well-being framework. These were discussed and agreed upon in a series of meetings with the government team, which included a specialist ergonomist, project manager specialising in workplace health and well-being, and senior leaders.

### 4.1. Adaptions to program delivery

One of the primary requirements was for the program to be able to be delivered with no/low cost for workplaces. Therefore, one of the key adaptions was the transferral of the administration of the program delivery and evaluation from the research team to a workplace champion (identified by the workplace). To facilitate this, a comprehensive, stand-alone, free toolkit was developed to provide workplace champions with a guide and resources to facilitate organisational buy in, and support delivery and evaluation of the program in their workplace. An online web-based format for the toolkit was chosen as it allowed for: increased reach of the program; rapid communication and evidence updates; integration of multi-media platforms; and, was a cost-effective mode of delivery. The toolkit follows a step-by-step process and uses a ‘train the champion’ approach.

### 4.2. Adaptions to program content

The materials and protocols from Stand Up Australia, alongside other evidence based research and health promotion materials and models, were used to inform the toolkit materials. Materials were adapted to follow five steps, consistent with existing guides [30]: Step 1: Getting support from management (i.e. presenting the business case and formalising commitment); Step 2: Needs assessment (i.e. assessment of workplace environment and staff health, behaviour, knowledge and attitudes prior to program implementation); Step 3: Preparing for the program (i.e. establishing a workplace wellbeing committee and running an all-of-staff information and consultation workshop to brainstorm and decide on three top stand up, sit less, move more strategies to implement in their workplace); Step 4: Putting it into practice (i.e. setting an action plan and launching the program); and, Step 5: Evaluation (assessment of program implementation; repeat of the staff survey and workplace audit).

Tools to support each of these steps (e.g. business case templates, sample policies, surveys, checklists, action plans) were developed. Other key adaptions from the original materials included the modification of wording to be less researcher oriented, more consumer friendly and understandable at the general population level, and the increased use of graphics to convey the intervention messages and goals of the program. Videos (all <10 minutes) were created as a tool for promoting buy in, raising awareness of the program and the key messages of stand up, sit less, move more, and to promote staff discussion (facilitated by the workplace champion). The evaluation framework used in the precursor trial was shortened in length and scope to be feasible for implementation without researcher input, resulting in the creation of an online, self-completion questionnaire. In addition, a component addressing the business case for reducing sitting in the workplace (not required previously in the research context), was developed. This component, which was based on the business case fact sheet developed by Comcare in 2012 [31], provides background evidence about the impacts of too much sitting, the return on investment for workplaces for supporting a healthy workplace, and suggests cost effective solutions to reduce the risks associated with too much sitting.

## 5. Discussion

This paper described the first phase of the translation of an evidence-based intervention – Stand Up Australia – into practice. Key elements for translation included: development of a research-government partnership leading to funding for, and collaborative adaption of, the original program for widescale uptake; and, the integration of program buy in, delivery and evaluation systems within the online toolkit. In subsequent phases, pilot testing in a range of workplaces will provide key insights into the success of initial adaptations with regard to feasibility of implementation, as well the effectiveness of the program on awareness, culture, and behaviour change in different work environments and job roles. This feedback and evaluation will inform Phase 3 (toolkit optimisation). This staged process of translation allows for appropriate time and testing, with further adaptations informed by the practice based evidence generated. The online format is particularly well-suited for this ongoing and evidence-driven optimisation. In conclusion, strong research-government partnerships and a supportive funding climate were critical elements for achieving the first stage of translation of the evidence-based Stand Up Australia program. Maintaining and expanding these partnerships will be vital to ensure success through the next stages of translation.
